# Morroniside regulates hair growth and cycle transition via activation of the Wnt/β-catenin signaling pathway

**DOI:** 10.1038/s41598-018-32138-2

**Published:** 2018-09-13

**Authors:** Lijuan Zhou, Han Wang, Jing Jing, Lijuan Yu, Xianjie Wu, Zhongfa Lu

**Affiliations:** Department of Dermatology, The Second Affiliated Hospital, School of Medicine, Zhejiang University, Hangzhou, China

## Abstract

Hair loss is characterized by a shortened hair anagen phase and hair follicles (HF) miniaturization. Morroniside is the most abundant iridoid glycoside extracted from *Cornus officinalis* and has various bioactivities in different cell functions and tissue regeneration. In this study, we investigated the effects and the underlying mechanism of morroniside on hair growth and regulation of HF cycle transition. Morroniside treatment significantly enhanced outer root sheath cell (ORSC) proliferation and migration *in vitro*. Additionally, morroniside upregulated Wnt10b, β-catenin and lef1. The enhanced ORSC proliferation and migration due to morroniside treatment were partly rescued by a Wnt/β-catenin signaling inhibitor, DKK1. Furthermore, in a hair-induced mouse model, morroniside injection accelerated the onset of anagen and delayed HF catagen, as shown by histological examination. Immunohistochemical analyses revealed that Wnt/β-catenin signaling pathway expression was upregulated in the HFs. These findings suggest that morroniside regulates HF growth and development partly through the Wnt/β-catenin signaling pathway and may be a potential treatment for hair loss.

## Introduction

Hair follicles (HFs) have a unique capacity to undergo growth (anagen), regression (catagen) and rest (telogen) before regenerating themselves and dynamically restarting the cycle^1^. Premature anagen-to-catagen transition induces hair growth inhibition and miniaturization^2^. Hair loss influences the appearance and psychological health of a high proportion of men and women^3^. Androgenic alopecia (AGA), the most common type of hair loss, is characterized by shortened anagen phase or delayed telogen-to-anagen transition and miniaturization of HFs^4^. Finasteride and minoxidil, two FDA-approved drugs and the most widely used alopecia treatments, are available. However, the effects of minoxidil are transient and may cause contact dermatitis or hypertrichosis, and finasteride usage is contraindicated in women^5^. Therefore, more efficient and safe treatments are urgently needed^6^.

**Figure 1:**
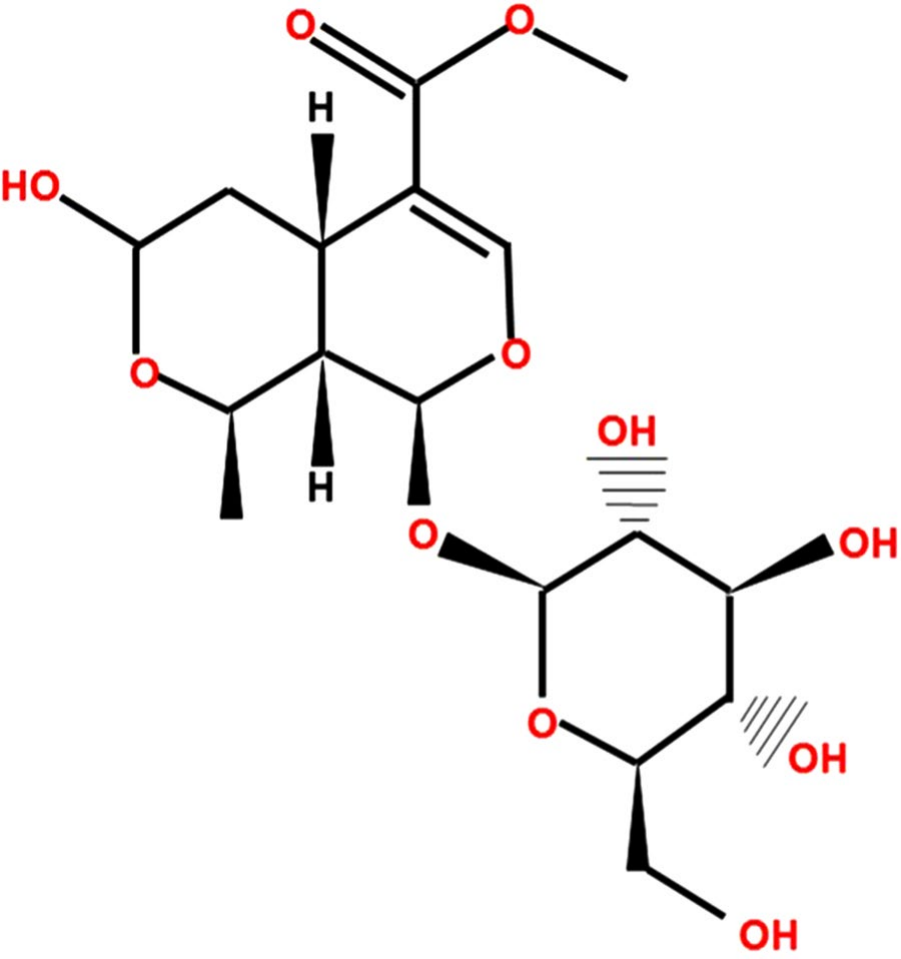
Chemical structure of morroniside.

Recently, traditional Chinese medicine extracts or monomers have been shown to have potent effects in promoting hair cell function, improving hair follicle miniaturization, preventing hair loss and significantly stimulating hair regrowth^7^. Morroniside is one of the most abundant iridoid glycosides extracted from *Cornus officinalis* (Fig. 1), which is prized as one of the most widely applied vegetable drugs in China^8^. Previous studies have demonstrated that morroniside exhibits many bioactivities, including protecting cells against apoptosis, promoting cell proliferation and facilitating tissue regeneration^9^. Hu *et al*. reported that morroniside promoted bone marrow mesenchymal stem cell (SC) proliferation through secreted factors, extracellular matrices and cellular adhesion molecules^10^. Li *et al*. demonstrated that morroniside directly promoted MC3T3-E1 cell proliferation, inhibited apoptosis and reduced bone resorption^11^. Xu *et al*. found that morroniside had protective effects on human umbilical vein endothelial cells for diabetic angiopathies, strongly enhanced endothelial progenitor cell proliferation and improved microvascular function after cerebral ischemia^12^. However, the effect of morroniside on HF growth and cycling regulation has not been investigated.

The Wnt/β-catenin signaling pathway is one of the most important signaling pathways for HF development^13–15^. After activation in adult tissues, β-catenin, the key transducer of the Wnt/β-catenin signaling pathway, accumulates in the cytoplasm and translocates to the nucleus, where it dimerizes with members of the lymphoid enhancer factor/T-cell factor (LEF/TCF) family of transcription factors^16^. This complex regulates downstream target gene transcription to control cell functions, such as proliferation and migration^15^. Previous studies have shown that Wnt signaling is critical for hair growth and development^17,18^. Wnt/β-catenin signaling is a master regulator of hair cell (such as hair matrix cell, outer root sheath cell (ORSC) and derma papilla cell) proliferation and migration and promotes cell-cell adhesion^16,19^. In addition, Wnt/β-catenin signaling is a key player in inducing the onset of anagen and maintaining the cycling transition during the initiation and regeneration of HFs^20,21^. Moreover, Wnt signaling promotes angiogenesis and plays an essential role in the morphogenesis of HFs^22^. Thus, Wnt/β-catenin signaling is crucial for HF regulation and could be a potential target for hair loss prevention and treatment. Additionally, morroniside and Wnt signaling are closely related. Sun *et al*. demonstrated that morroniside enhanced neural SC proliferation and promoted neurogenesis via the Wnt/β-catenin signaling pathway^23^.

In this study, we first investigated the function of the natural compound morroniside in HF growth and hair cycle regulation. We found that the morroniside enhanced the proliferation and migration of ORSCs *in vitro*. Moreover, morroniside induced anagen while delaying catagen, producing longer hair shafts and larger bulges in treated mice compared to controls. We also demonstrated that these *in vitro* and *in vivo* effects were mediated by the Wnt/β-catenin signaling pathway. These data highlight a novel role for morroniside in the regulation of HF growth and development and provide a potential strategy for the treatment of hair loss.

## Results

### Morroniside stimulated ORSC proliferation and increased ORSCs in the S, G2 and S/G1 phases

**Figure 2:**
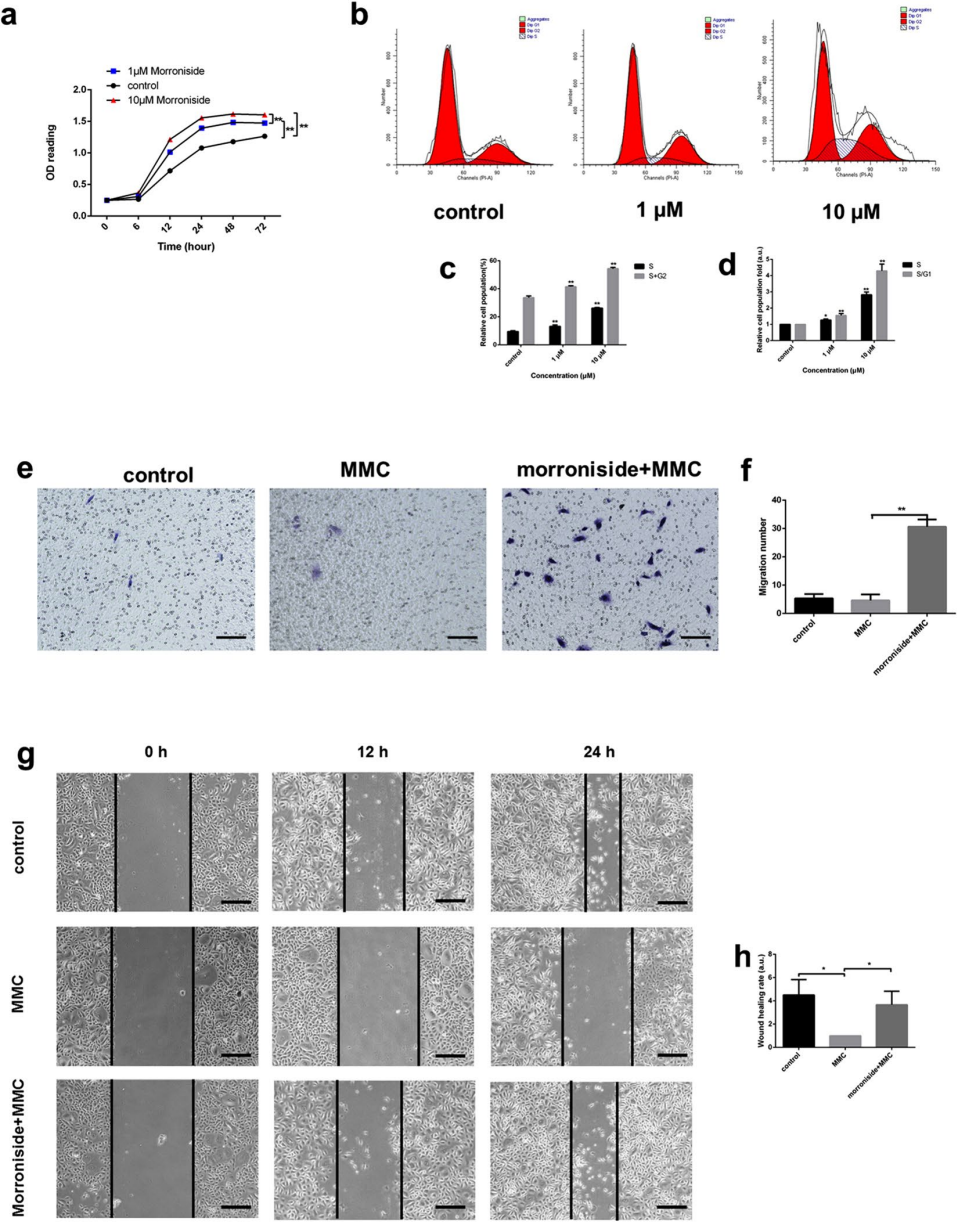
Morroniside treatment stimulated human ORSC proliferation and migration and increased cells in S and S/G1 phases *in vitro*. (**a)** Morroniside increased proliferation of ORSCs in a dose-dependent manner, as shown by MTS assays. (**b**,**c**) Distributions of ORSCs in G1, S, and G2 phases in the presence or absence of morroniside were detected by flow cytometry. (**d**) Fold changes in the fractions of cells in S and S/G1 phases with or without morroniside treatment. (**e**,**f**) Transwell assays of ORSCs with or without morroniside treatment. (**g**,**h**) Wound scratch assays of ORSCs cultured with or without morroniside treatment. a.u., arbitrary units. MMC, mitomycin C. Bars represent the mean ± SD. *P < 0.05, **P < 0.01.

The morroniside-treated group showed no significant change on apoptosis compared with the control group after 24 h and 72 h (in Supplementary Fig. S2). When administered at concentrations of 1 and 10 µM, morroniside (Fig. 1) promoted ORSC proliferation in a concentration-dependent manner compared to that of the untreated control group (Fig. 2a). 1 and 10 µM morroniside significantly increased proliferation by 1.16-fold (P < 0.01) and 1.3-fold (P < 0.01) at 72 h, respectively. At a concentration of 10 µM, morroniside showed a more powerful effect on ORSCs proliferation compared with the control group or with the group treated with 1 µM morroniside. The cell cycle status of ORSCs in the presence or absence of morroniside was evaluated by PI staining and flow cytometry (Fig. 2b). The data showed that 40.82% (P < 0.01) and 53.40% (P < 0.01) of ORSCs entered into the S and G2 phases with 1 and 10 µM morroniside, respectively, whereas the vehicle-treated cells showed only approximately 33.39% of the cells present in the S and G2 phases (Fig. 2c). Morroniside treatment caused 1.26- (P < 0.05) and 2.67-fold (P < 0.01) increases in the size of the S phase fraction and 1.42- (P < 0.01) and 3.82-fold (P < 0.01) increases in that of the S/G1 fraction at concentrations of 1 µM and 10 µM, respectively (Fig. 2d). These results provide further evidence that morroniside stimulated ORSC proliferation.

### Morroniside stimulated ORSC migration

We further investigated whether morroniside stimulation leads to increased ORSCs migration and “wound” closure using an *in vitro* transwell assay and scratch assay. To minimize the influence of morroniside on ORSC proliferation, we pre-treated cells with 10 µg/ml mitomycin C (MMC) for 2 h^24^. Morroniside treatment at a concentration of 10 µM increased ORSC migration relative to that of untreated cells (Fig. 2e,f). In addition, the migration rate of the cells in the wound healing assay showed that 10 µM morroniside enhanced ORSC motility (Fig. 2g,h).

### Morroniside activated the Wnt/β-catenin signaling pathway

**Figure 3:**
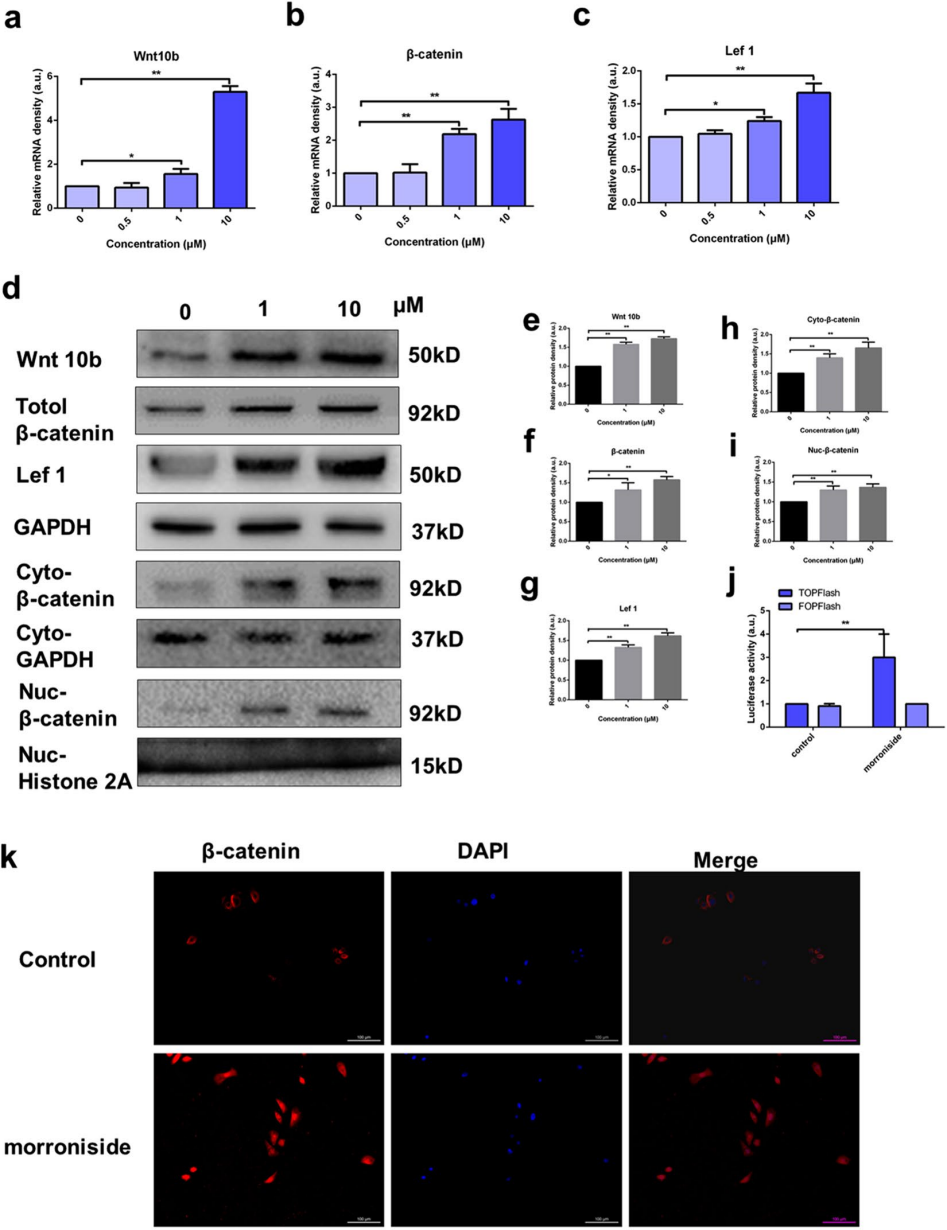
Morroniside activated the Wnt/β-catenin signaling pathway. (**a**–**c**) Relative mRNA expression levels of Wnt10b, β-catenin, and lef1 in human ORSCs, determined by RT-PCR. (**d**) Wnt10b, β-catenin (total, cytoplasmic and nuclear), and lef1 protein expression levels in human ORSCs, detected by western blotting. The grouping of blots was cropped from different gels. GAPDH was used as the control for total and cytoplasmic proteins, whereas histone 2A was used as the control for the nuclear proteins. (**e**–**i**) Quantitative analysis of Wnt10b, β-catenin (total, cytoplasmic and nuclear), and lef1 protein levels. (**j**) TOPFlash reporter activity induction in human ORSCs was detected by TOPFlash reporter assay. a.u., arbitrary units. (**k**) β-catenin is shown in red, and nuclei counterstained with DAPI (blue). Merged images indicate the expression and location of β-catenin. *P < 0.05, **P < 0.01.

To confirm the above results suggesting a role for Wnt/β-catenin signaling, we measured the expression levels of Wnt10b, β-catenin and lef1 by reverse transcription (RT)-PCR, western blotting analysis and immunofluorescence analysis after morroniside treatment for 24 h. The results of the RT-PCR analyses revealed increased expression of Wnt10b, β-catenin and lef1 in the morronside-treated group (Fig. 3a–c). According to Fig. 3d–i, compared with the control group, the protein expression of Wnt 10b, β-catenin (total, nuclear and cytoplasmic), and lef1 increased in the morroniside-treated group. The effect of morroniside on β-catenin activity was evaluated by TOPFlash reporter assay. Morroniside significantly induced the TOPFlash reporter activity of ORSCs without any effects on FOPFalsh activity, which served as a negative control (Fig. 3j).

Immunofluorescence analysis showed increased accumulation of β-catenin in the cytoplasm, and β-catenin translocated into the nucleus in the morronside-treated group compared with the control group (Fig. 3k).

### Increased ORSC proliferation and migration of morroniside were partially downregulated by a Wnt/β-catenin inhibitor

**Figure 4:**
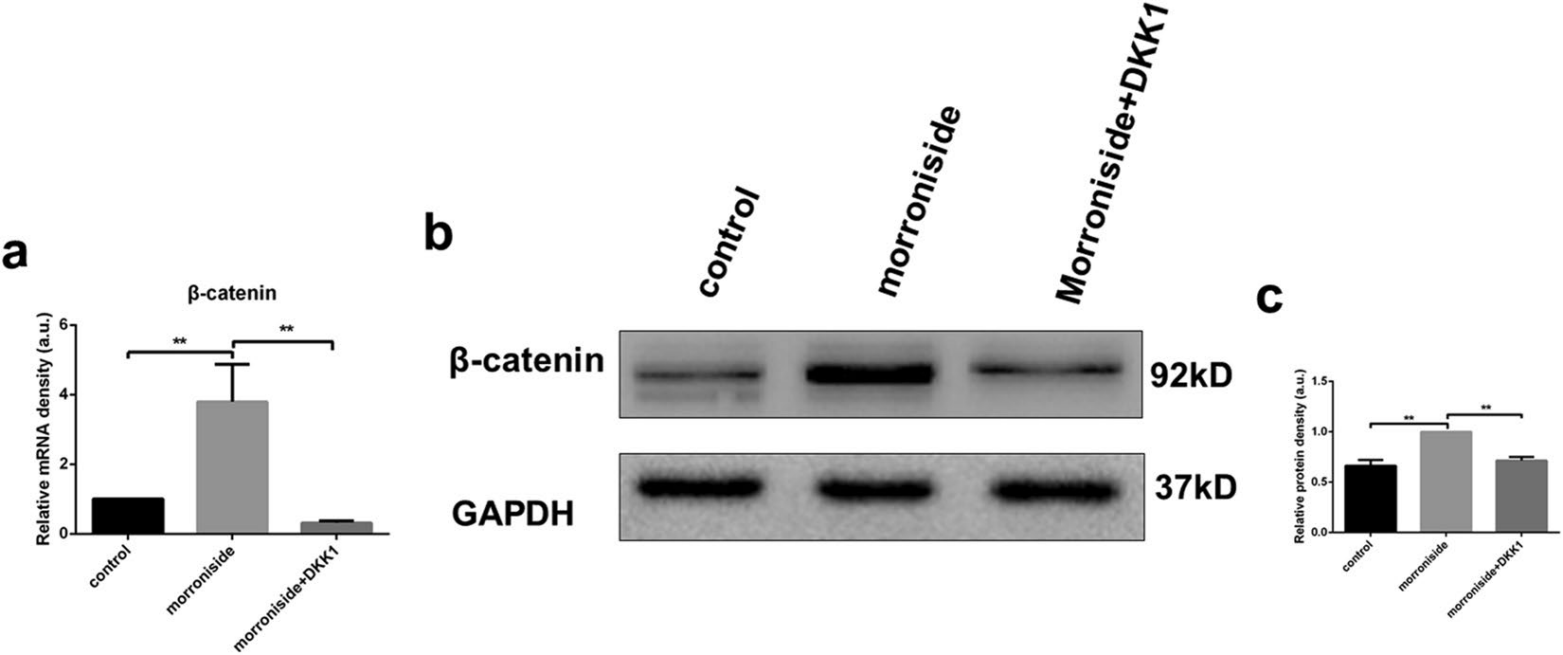
The increased mRNA and protein levels of β-catenin were partially reversed by addition of a Wnt/β-catenin signaling inhibitor (DKK1). (**a**) The β-catenin mRNA levels in control, morroniside and morronside +DKK1 groups were determined by RT-PCR after treatment for 24 h. (**b**) Total β-catenin protein levels in control, morroniside and morronside +DKK1 groups were determined by western blotting. The grouping of blots was cropped from the same gel. (**c**) Quantitative analysis of β-catenin protein levels in control, morroniside and morroniside +DKK1 groups. a.u., arbitrary units.

**Figure 5:**
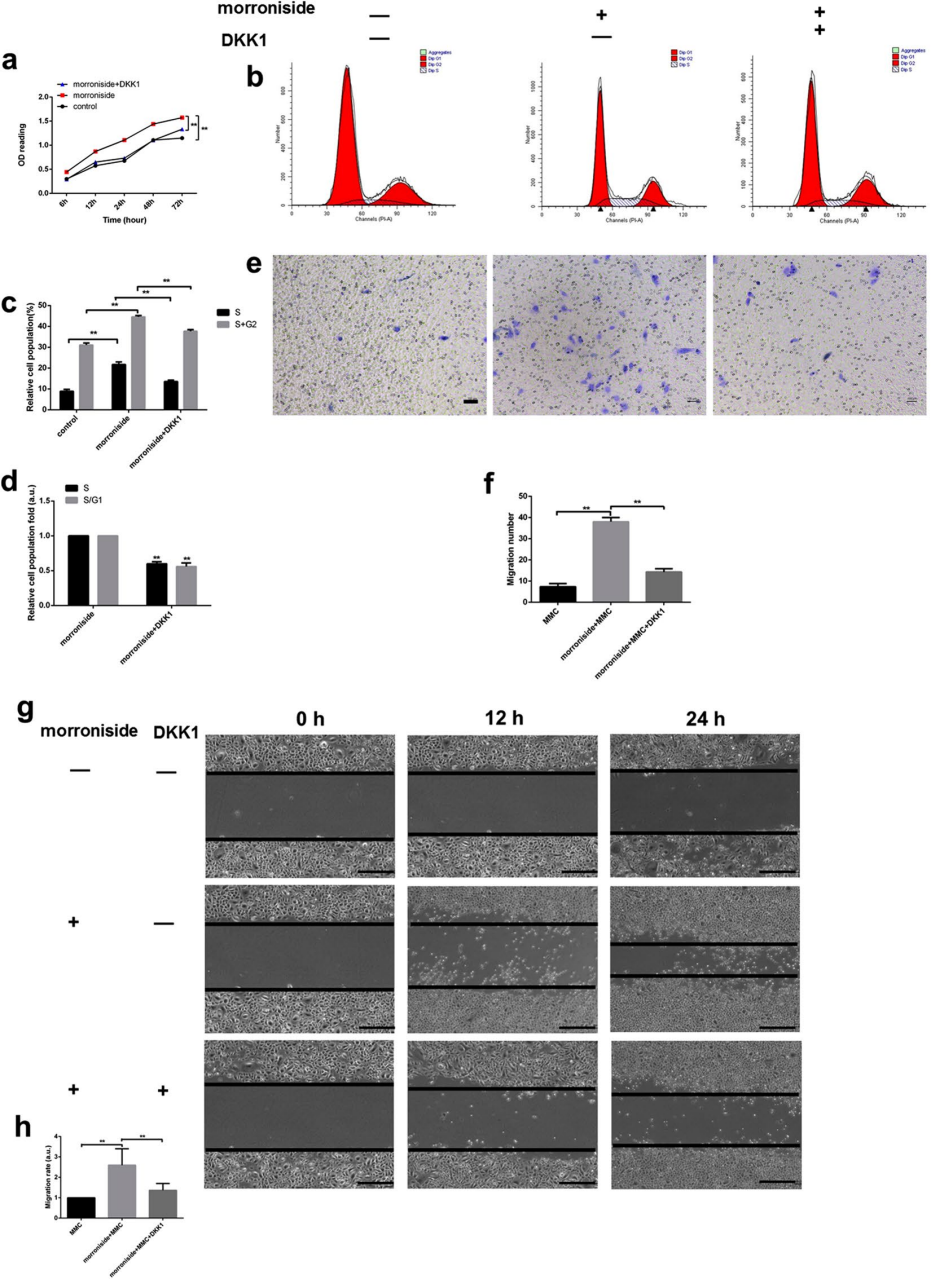
The increased proliferation and migration caused by morroniside were partially rescued by addition of a Wnt/β-catenin signaling inhibitor (DKK1). (**a)** The proliferation of ORSCs in control, morroniside and morronside +DKK1 groups was measured by MTS assays at 0, 6, 12, 24, 48, and 72 h, respectively. (**b**,**c**) Distributions of ORSCs in G1, S, and G2 phases in control, morroniside and morronside +DKK1 groups were detected by flow cytometry. (**d**) Fold changes in the fractions of cells in S and S/G1 phases in control, morroniside and morronside +DKK1 groups. (**e**,**f**) Transwell assays of ORSCs in control, morroniside and morronside +DKK1 groups. (**g**,**h**) Wound scratch assays of ORSCs in control, morroniside and morronside +DKK1 groups. a.u., arbitrary units.

To confirm the involvement of the Wnt/β-catenin signaling pathway, we examined the effect of Wnt/β-catenin inhibition on the proliferation and migration of ORSCs in the morroniside-treated group. After the addition of DKK1 for 24 h, the mRNA level of β-catenin and protein level of total β-catenin were strongly decreased compared with the β-catenin level in the morroniside-treated ORSCs without DKK1 (Fig. 4a–c). Moreover, inhibition of Wnt/β-catenin signaling partially reversed the increased proliferation of ORSCs (Fig. 5a). The data showed that 38.54% (P < 0.01) of ORSCs entered the S and G2 phases following treatment with 10 µM morroniside and DKK1, and the morroniside-treated cells showed approximately 43.73% of cells in the S and G2 phases (Fig. 5b,c). DKK1 treatment caused 0.63-fold (P < 0.01) decreases in the size of the S phase fraction and 0.58-fold (P < 0.01) decreases in that of the S/G1 fraction compared with those of the 10 µM morroniside treatment (Fig. 5d). In addition, inhibition of Wnt/β-catenin signaling partially reversed the increased migration of ORSCs (Fig. 5e–h).

### Local injection of morroniside accelerated telogen HF entry into anagen

**Figure 6:**
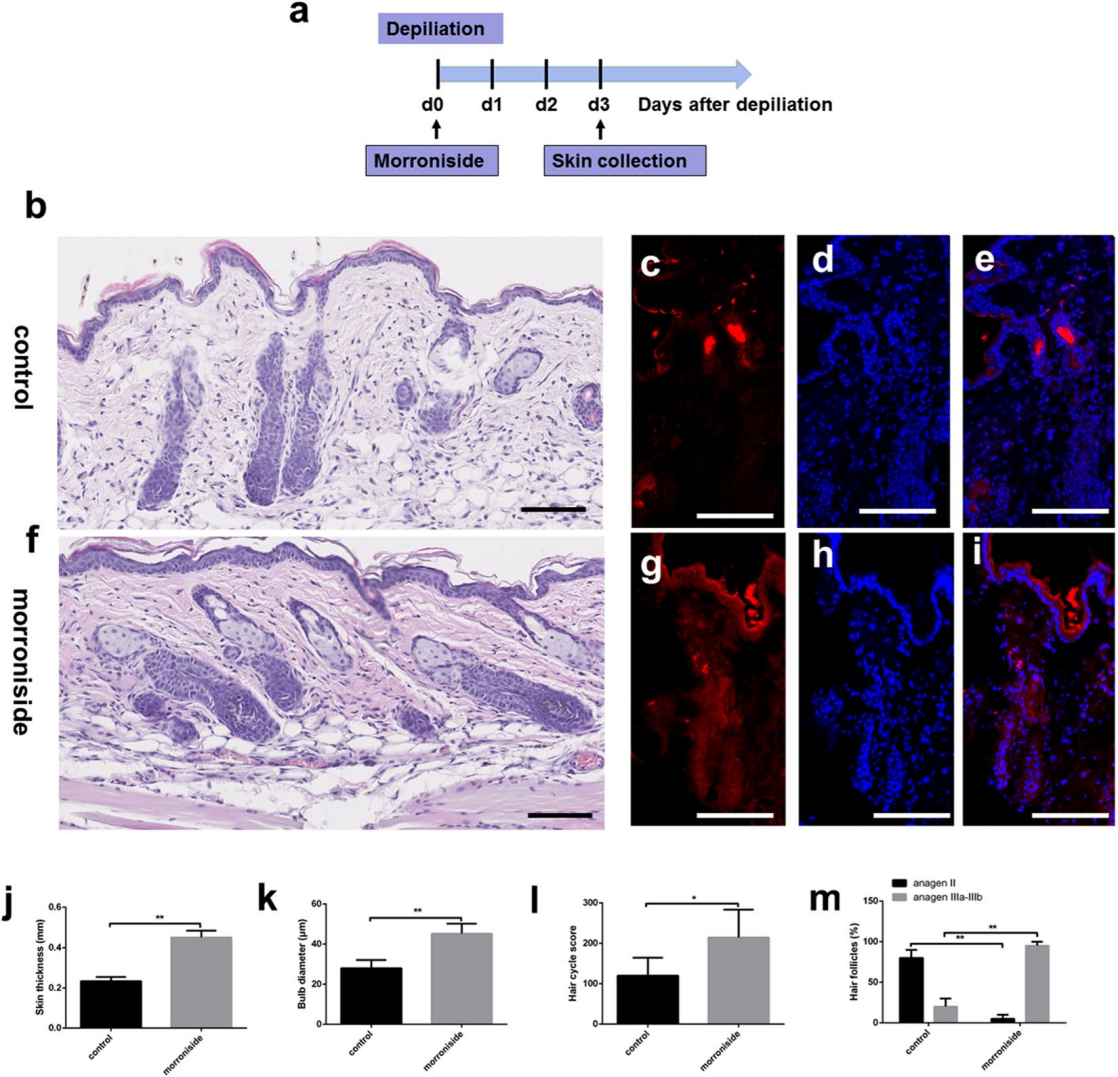
Local injection of morroniside accelerated transition of mouse HFs from telogen to anagen. Morroniside (100 µM in 100 µL) was cutaneously injected into mouse HFs on p.d. 0 (100 μL over 3 days, total 300 μL). Mice were sacrificed on p.d. 3. Results represent the mean ± SD (n = 6 per group). *P < 0.05, **P < 0.01. (**a**) Experimental time course; (**b**–**e**) Control group. (**f**–**i**) Morroniside group. (**c**–**e**,**g**–**i**) Ki67 is shown in red, and nuclei are counterstained with DAPI (blue). Merged images indicate the expression and location of Ki67. (**j**) Skin thickness. (**k**) Bulb diameter. (**l**) Hair cycle score. (**m**) Percentage of HFs. Scale bar: 100 μm.

A histological analysis revealed that injection of morroniside (100 µM) into the telogen dorsal skin of mice accelerated entry into anagen phase. The morphology of morroniside-treated HFs was characteristic of anagen phase, with thicker skin and larger hair bulges surrounded by increased melanin and more bulbs in the subcutis (Fig. 6f). In contrast, skin treated with phosphate-buffered saline (PBS) exhibited the morphology of the earlier anagen phase, with most hair bulbs present at the dermis-subcutis border (Fig. 6b). In addition, we performed Ki67 (proliferation marker) immunofluorescence (Fig. 6c–e,g–i). HF cells in the morroniside-treated group had more proliferating (Ki67-positive) cells than the control group, especially in the regions of proximal hair matrix and the epidermis. According to a previous study^25,26^, we assigned scores of 100, 200, and 300 for anagen I–IIIa, anagen IIIb–IIIc, and anagen IV–VI HFs, respectively. Skin thickness and bulb diameter (Fig. 6j,k) as well as hair cycle score (HCS) (Fig. 6l) were higher in the morroniside-treated group than the control group. HFs in morroniside-treated sites were in the later anagen phase; most were in phase IIIb of anagen, and some were in anagen IV phase, with some melanin around the bulbs, compared to the early anagen phase (anagen II–IIIa) in the control. Over 80% of PBS-treated HFs remained in early anagen phase, while nearly 90% of HFs in morroniside-treated skin were in anagen IIIb to V (Fig. 6m). These results suggested that morroniside accelerated hair cycling from telogen to anagen.

### Morroniside injection into mouse HFs in anagen delayed entry into catagen

**Figure 7:**
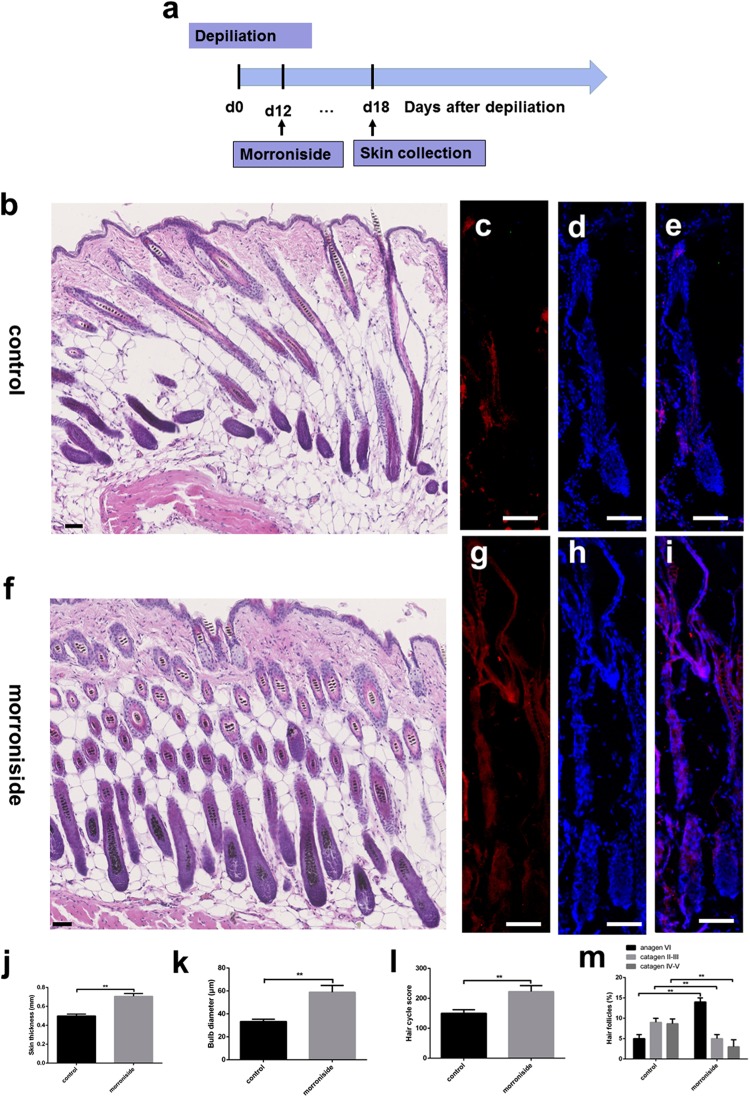
Injection of morroniside into HFs delayed transition of mouse HFs from anagen to catagen. Morroniside (100 μM in 100 μL) was cutaneously injected into mouse HFs on p.d. 12 (100 μL over 6 days, total 600 μL). Mice were sacrificed on p.d. 18. Results represent the mean ± SD (n = 6 per group). *P < 0.05, **P < 0.01. (**a**) Experimental time course. (**b**–**e**) Control group. (**f**–**i**) Morroniside group. (**c**–**e**,**g**–**i**) Ki67 is shown in red, and nuclei are counterstained with DAPI (blue). Merged images indicate the expression and location of Ki67. (**j**) Skin thickness. (**k**) Bulb diameter. (**l**) HCS. (**m**) HFs (%). Scale bar: 100 μm.

To investigate the effect of morroniside treatment on the anagen-catagen transition of the HF cycle, we divided the mice into two groups that were treated with morroniside (100 µM) or vehicle. A histomorphometric analysis of H&E-stained tissue sections revealed that the morphology of HFs with morroniside treatment (Fig. 7f) was that of anagen VI phase, with a significantly larger bulb diameter and thicker skin compared to those of the vehicle-treated group (Fig. 7b). Meanwhile, control mice had a narrower dermal papilla and thinner skin than the morroniside group (Fig. 7j,k). Ki67 immunofluorescence (Fig. 7c–e,g–i) showed that HF cells in the morroniside-treated group included more Ki67-positive (proliferating) cells than the control group, especially in the regions of the epidermis, ORS and hair bulbs. We also calculated HCS and HF (%) to more accurately determine the hair cycle stage. According to a previous study^25,26^, we assigned scores of 100, 200, and 300 for anagen VI, catagen II–III, and catagen IV–V HFs, respectively. The total HCS was lower in the morroniside group than in the control group (Fig. 7l); 10% of HFs in the former had entered catagen IV–VI stage vs. approximately 20% in the latter (Fig. 7m). These results indicate that morroniside treatment in mouse skin could delay catagen progression.

### Upregulated Wnt/β-catenin signaling expression by morroniside in HFs in the telogen-to-anagen and anagen-to-catagen transition stages

**Figure 8:**
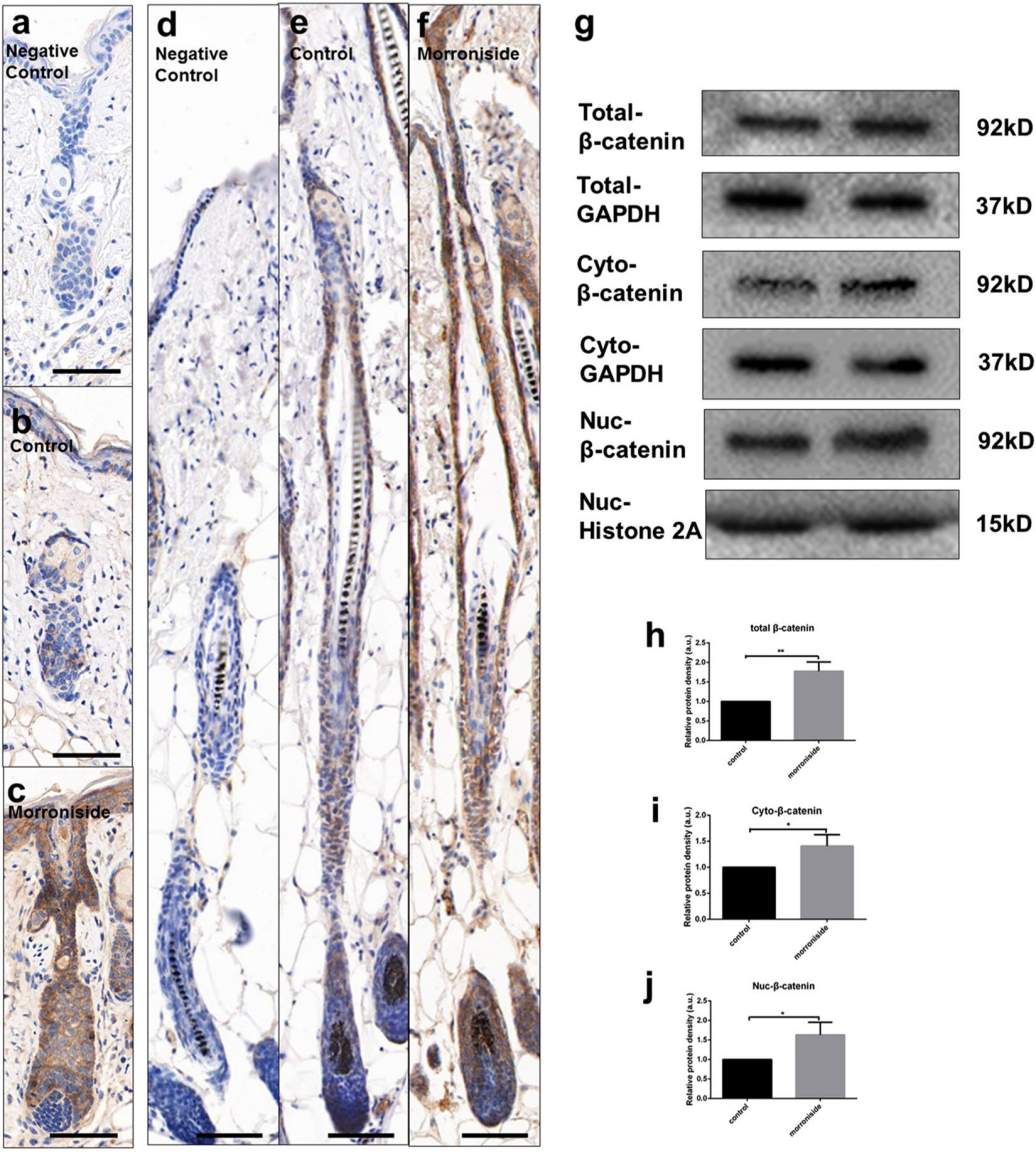
Upregulation of β-catenin expression in morroniside-treated groups. β-catenin protein was detected in the dorsal skin of mice by immunohistochemistry on p.d. 3 and 18. (**a**,**d**) Negative control. (**b**,**e**) β-catenin expression in the PBS-treated group on day 3 (**b**) and day 18 (**e**). (**c**,**f**) β-catenin expression in the morroniside-treated group on day 3 (**c**) and day 18 (**f**). (**g**) Total, cytoplasmic and nuclear β-catenin protein expression levels in mice HFs with or without morroniside treatment, detected by western blotting. The grouping of blots was cropped from different gels. GAPDH was used as the control of the total and cytoplasmic proteins, whereas histone 2A was used as the control for the nuclear proteins. (**h**–**j**) Quantitative analysis of β-catenin (total, cytoplasmic and nuclear) protein level. Scale bar: 50 μm.

To further evaluate whether the expression of Wnt/β-catenin signaling was also influenced by morroniside (100 µM) in HF cycling transition, we performed IHC analysis of murine HFs, and the results revealed that β-catenin staining in the morroniside-treated group was more intense than that in the control group throughout the epidermis, ORS and hair matrix (Fig. 8a–f). During telogen-to-anagen transition, in the morroniside-treated groups, β-catenin staining was localized mainly at the epidermis, ORS and the hair matrix (Fig. 8c). The distribution of β-catenin in the control groups was primarily observed in the epidermis and nearly disappeared in the ORS and hair matrix (Fig. 8b). During the anagen-to-catagen transition, in the morroniside-treated groups, β-catenin was strongly expressed at the epidermis, ORS and hair matrix (Fig. 8f). β-catenin in the control groups showed weak staining at the epidermis and ORS (Fig. 8e). Moreover, the nuclear β-catenin staining was more obvious in hair bulb cells in the morroniside group (Fig. 8c,f) than the control group (Fig. 8b,e). In addition, we extracted and analyzed the tissue nuclear and cytoplasmic β-catenin protein expression (Fig. 8g) and found that the expression of the total, cytoplasmic and nuclear β-catenin expression level were increased in the morroniside-treated groups compared with the control groups (Fig. 8h–j). Taken together, these data indicated that morroniside promotes hair growth partly by upregulating β-catenin.

## Discussion

To the best of our knowledge, this is the first study to explore the effect of morroniside on hair growth and cycling transition regulation and its potential mechanisms. We found that morroniside significantly accelerated the proliferation and migration of ORSCs partly via the Wnt/β-catenin signaling pathway *in vitro*. *In vivo*, morroniside prolonged anagen phase by inducing anagen while delaying catagen, and IHC analysis results showed that β-catenin was strongly stained. These findings indicated that morroniside regulates HF growth and development partly through activation of the canonical Wnt/β-catenin signaling pathway and highlight a novel strategy for the treatment of hair loss.

Here, we isolated and cultured human scalp ORSCs by using a two-step enzymatic digestion method^27,28^. ORSCs play a pivotal role in hair and epidermis biology^20^. First, the proliferation and migration of ORSCs contributes to hair shaft formation^20^. Second, ORSCs, which can secrete numerous signal molecules, play critical roles in HF epithelial-mesenchymal interaction and further regulate the HFSC niche and HF development microenvironment^29,30^. Third, ORSCs have a high proliferation capacity, playing an important role in anagen initiation and hair regeneration^31^. Thus, ORSCs play a critical role in the maintenance and development of hair growth and cycling transition^27^. In the present study, MTS-based assays and cell cycle analysis showed that morroniside had positive, dose-dependent effects on ORSC proliferation and induced G1-S cell cycle progression of ORSCs; in addition, transwell assays and “wound healing” assays showed that morroniside enhanced ORSC migration. These data elucidate the effects of morroniside on HF development and growth.

To confirm the positive effect of morroniside on HF growth and cycling transition, we further investigated the function of morroniside in a mouse model. Hair undergoes cyclic transformations from the rapid growth stage (anagen) to regression stage (catagen) and then to relative quiescence (telogen)^27^. The onset of anagen in HFs recapitulates HF growth and development, with hair cell proliferation and new long hair shaft formation; conversely, HFs stop cycling and hair is lost during the catagen phase^32^. Increased cycling of HFs out of anagen and into catagen results in increased numbers of shed hairs^33^. AGA, which is the most common type of hair loss, is characterized by shortened anagen phase and HF miniaturization^4^. In the present study, local injection of morroniside (100 µM) accelerated anagen and delayed catagen, which indicated that morroniside elongates the anagen phase and regulates hair cycling transition, promoting hair growth. Presently, the most widely used drugs in hair loss treatment, such as finasteride and minoxidil, induce and prolong the anagen phase and convert vellus follicles into terminal follicles^4,32^. Therefore, morroniside may have a potential role in treating abnormal hair cycle transition-induced alopecia by prolonging the hair anagen phase.

The Wnt/β-catenin signaling pathway is an essential signaling pathway required for HF growth and cycling transition^34,35^. In canonical Wnt signaling, Wnt ligands bind to LRP5/6 coreceptors and Frizzled receptors and inactivate the β-catenin destruction complex, leading to β-catenin accumulation and translocation into the nucleus. There, β-catenin regulates target genes by interacting with Tcf/Lef transcription factors^16^. DKK1, as a potent Wnt signaling antagonist, binds to LRP5/6 and blocks the Fz-LRP association, which subsequently induces specific inhibition of the Wnt/β-catenin signaling pathway^36^. In the present study, we observed that morroniside increased Wnt/β-catenin signaling in cultured human ORSCs, and immunofluorescence analysis confirmed these findings. Furthermore, the increased proliferation and migration of ORSCs by morroniside were partially reversed by an inhibitor of Wnt/β-catenin signaling (DKK1). G1-S transition is critical for cell cycle progression, and DKK1 notably reduced morroniside-mediated cell proliferation and decreased G1/S transition. These findings indicated that morroniside promotes ORSC proliferation and migration partly via activation of the Wnt/β-catenin signaling pathway.

Previous studies have already demonstrated that morroniside not only stimulated various types of cell proliferation and migration but also promoted tissue regeneration^8,23^. Morroniside has several obvious advantages as a potential therapy. First, morroniside is safe. This compound is the most active component in *C*. *officinalis*, which is a food and medical plant classified by the Ministry of Health of the People’s Republic of China and has been widely used clinically for thousands of years^37^. Moreover, morroniside, as a plant extract, did not alter xenobiotic metabolizing enzymes and transporters^38^. In addition, previous studies have shown that morroniside has no toxicity in various cell types and no obvious adverse effects in animals^37,39^. Second, morroniside is a convenient and stable compound. It can be dissolved in water or PBS for use and easily transported and preserved^37^. Third, morroniside is cheap and could be easily produced. This compound is extracted and purified from *Corni Fructus*, which is planted in large quantities^38^. The extraction and purification process has been developed, and morroniside purity was determined to be 98.5% through high-performance liquid chromatography^40^. More importantly, compared with finasteride, the most widely used drug in AGA, which suppresses the anagen-to-catagen phase transition in HFs^5,41^, morroniside could not only delay anagen transition into catagen but also initiate telogen into anagen. Thus, morroniside has the potential to be a promising drug to treat anagen-reduced hair loss in the future.

Many studies have demonstrated a relationship between morroniside and cell function or tissue formation and regeneration^9,23^. However, this is the first study to demonstrate the impact of morroniside on hair growth and cycling transition. Unfortunately, the mechanism of Wnt/β-catenin signaling activation by morroniside in animals is not fully clarified. Other signaling pathways need to be explored to determine their potential involvement in the hair growth and cycling transition regulation by morroniside in future studies.

In conclusion, the results of the present study provide the first evidence that morroniside may contribute to the regulation of HF growth and cycling regulation through the Wnt/β-catenin signaling pathway. We showed that morroniside exerted a significant stimulatory effect on ORSC proliferation and migration compared to the vehicle. Moreover, morroniside injection during HF telogen accelerated the onset of anagen, whereas injection during anagen delayed catagen. Thus, morroniside may represent a potential treatment for hair loss.

## Materials and Methods

All methods were performed in accordance with the relevant guidelines and regulations.

### Isolation and culture of normal ORSCs from normal human scalp

The Zhejiang University Institutional Review Board approved the use of ORSCs. Healthy human scalp specimens (n = 20; from 10 females and 10 males, age range: 18–50 years) were obtained with informed consent from subjects without systemic diseases who were undergoing cosmetic surgery. ORSCs were isolated from scalp HFs as previously described^27,42^. Briefly, the specimens were rinsed 3 times for 10 min with PBS and then cut into several small strips at the derma-subcutaneous fat border using clippers and incubated in 0.5% dispase overnight at 4 °C. HFs were plucked off, and the dermal papilla was cut off under microscope. HFs were incubated in 0.25% trypsin-EDTA at 37 °C for 15 min; the trypsin activity was then neutralized with fetal bovine serum (FBS). The ORSC suspension was washed and centrifuged twice at 800 × *g* for 5 min, resuspended in complete keratinocyte serum-free medium (KSFM), transferred to 25 ml flasks and cultured at 37 °C in a humidified atmosphere of 5% CO_2_. The study protocol was approved by the Zhejiang University School of Medicine Second Affiliated Hospital Institutional Review Board. We confirmed the expression of keratin 14 in our cultured human ORSCs by immunofluorescence (in Supplementary Fig. S1).

### Cell proliferation assay

For analysis of the effect of morroniside on the proliferation of ORSCs, the cells (1.5 × 10^4^ cells/well) were seeded in a 96-well plate. When grown to 70–80% confluence, ORSCs were treated with morroniside at 0, 1, and 10 µM. After 0, 6, 12, 24, 48, and 72 h, the ORSCs were treated with 3-(4,5-dimethylthiazol-2-yl)-5-(3-carboxymethoxyphenyl)-2-(4-sulfophenyl)-2H-tetrazolium (MTS) (Promega, Madison, WI, USA) solution for 30 min at 37 °C before absorbance was measured at 490 nm on an ELX808 microplate reader (BioTek, Winooski, VT, USA). This assay was repeated at least 3 times.

### Cell cycle and apoptosis analysis by flow cytometry

ORSCs were seeded in 25 cm^2^ culture dishes and treated with morroniside (0, 1, 10 µM) treatment for 24 h. After culture, ORSCs were digested to obtain a single-cell suspension with cold 80% precooled ethanol in PBS and stained with propidium iodide (PI)-RNase using a kit (BD Biosciences, San Jose, CA, USA) for 20 min in the dark according to the manufacturer’s protocol. For the apoptosis assay, ORSCs were stained with a FITC Annexin V Apoptosis Detection Kit I (BD Biosciences) according to the manufacturer’s protocol (in Supplementary Fig. S2).

### Proliferation inhibition with MMC

ORSCs were grown to confluence and treated with 10 µg/ml of MMC for 2 h. Afterwards, ORSCs were washed with PBS and incubated in culture medium. The transwell assay and scratch assay were performed in MMC-pretreated cells.

### Transwell migration assay

ORSC migration was evaluated with a transwell assay as previously described^28^, with some modifications. Briefly, 500 μl of defined SFM containing morroniside was added to the lower chamber of the transwell insert; a 200-μl volume of cell suspension (1 × 10^6^/ml) was added to the upper chamber. After culture for 24 h at 37 °C and 5% CO_2_, cells on the upper surface of the membrane were carefully wiped off, whereas those on the undersurface of the membrane were fixed with 4% paraformaldehyde for 15 min, stained with 2% crystal violet for 5 min, and then rinsed under running water. The number of migrated cells in five random fields was counted. The assay was repeated three times.

### RT-PCR

ORSCs were treated with 0, 1, and 10 µM morroniside, and total RNA was isolated using TRIzol reagent (Ambion, New York, NY, USA). Reverse transcription was carried out using a high-capacity RNA to cDNA kit (TaKaRa Bio, Otsu, Japan) according to the manufacturer’s instructions. RT-PCR was carried out with SYBR Green I Master Mix (TaKaRa). Sequences of the specific primers (Sangon Biotech, China) used for RT-PCR are listed in Table 1. PCR was performed for 40 cycles, and the relative target gene levels were calculated by the 2^−ΔΔCt^ method.

**Table 1:** Sequences of primers used for RT-PCR.

Gene Name	Primer	Sequence
GAPDH	Forward	5′-CTCACCGGATGCACCAATGTT-3′
	Reverse	5′-CGCGTTGCTCACAATGTTCAT-3′
Wnt 10b	Forward	5′-CATCCAGGCACGAATGCGA-3′
	Reverse	5′-CGGTTGTGGGTATCAATGAAGA-3′
β-catenin	Forward	5′-GCTGGTGACAGGGAAGACAT-3′
	Reverse	5′-CCATAGTGAAGGCGAACTGC-3′
Lef1	Forward	5′-CTTCCTTGGTGAACGAGTCTG-3′
	Reverse	5′-TCTGGATGCTTTCCGTCAT-3′

### Western blot analysis

Western blotting was performed as previously described^27,28^. Briefly, ORSCs were treated with 0, 1, and 10 µM morroniside for 24 h. Total cellular protein was extracted with lysis buffer (Beyotime Institute of Biotechnology, Beijing, China). To detect β-catenin, proteins in the cytoplasm and nucleus were extracted using the Nuclear and Cytoplasmic Protein Extraction Kit (Beyotime). Proteins were transferred to a polyvinylidene difluoride membrane (Millipore) after separation by SDS-PAGE. Then, the membrane was blocked in 7% non-fat milk for 2 h at room temperature, followed by overnight incubation at 4 °C with the following primary antibodies in TBST containing 5% bovine serum albumin: rabbit polyclonal anti-Wnt10b (1:2000; Abcam, Shanghai, China), rabbit monoclonal anti-β-catenin, and rabbit polyclonal anti-lef1 (1:1000; Cell Signaling Technology, Danvers, MA, USA). The blots were washed three times for 10 min with TBST and then incubated for 2 h with horseradish peroxidase-conjugated anti-rabbit IgG (1:5000; Jackson Laboratories, West Grove, PA, USA), followed by 3 washes for 10 min each with TBST. Immunoreactive bands were detected using an enhanced chemiluminescence system (Millipore). GAPDH (1:1000; Cell Signaling Technology) and Histone 2 A (1:5000; Proteintech) were respectively used as internal controls for cytoplasmic and nuclear proteins. Protein density was measured by a Bio-Rad XRS chemiluminescence detection system (Bio-Rad, USA). The blots shown are representative of at least three repeats.

### Immunofluorescence

ORSCs were cultured in a 12-well plate and treated with 0 and 10 µM morroniside for 24 h. ORSCs were fixed in 4% paraformaldehyde for 15 min, permeabilized with 0.1% Triton-100, and blocked with 15% FBS for 1.5 h. Fixed ORSCs were washed with PBS and incubated overnight at 4 °C with β-catenin (1:250; Cell Signaling Technology, Danvers, MA, USA). After washing, ORSCs were incubated with a fluorescence-conjugated secondary antibody (Jackson) for 2 h, and nuclei were stained with 4′,6-diamidino-2-phenylindole (Roche) for 5 min. β-catenin was detected using a fluorescence microscope (EU5888; Leica, Wetzlar, Germany).

### TOPFlash and FOPFlash reporter assay

ORSCs were seeded into 96-well dishes before transfection. At 70–80% confluence, TOPFlash plasmid (VT1592; YouBio) was transfected into each well with or without morroniside using Lipofectamine 3000 (Invitrogene). FOPFlash plasmid (VT1593; YouBio) was used as a control. The relative luciferase activities were determined through the Dual-Gloluciferase system (Promega, USA) according to the protocol.

### Experimental studies with morroniside

All C57BL/6 mice were supplied by the Academy of Medical Sciences of Zhejiang province. All the animal experiments were performed in accordance with the Animal Care and Use Committee guidelines of Zhejiang province. All the experimental procedures were approved by the Institutional Animal Care and Use Committee at Zhejiang University. Twenty four C57BL/6 mice (female, 7 to 8 weeks old, only mice in telogen phase could be used for this study) in 4 randomized groups (n = 6) were used to study the hair-promoting activity of morroniside. Anagen was induced by depilation in the mouse back skin, and all HFs in the depilated region were in exactly the same anagen phase as a spontaneous anagen development. Morronside or vehicle (sterile PBS) was injected into the depilated dorsal skin of C57BL/6 mice in depilation-induced telogen and anagen VI phases (post-depilation day [p.d.] 0 and 12, respectively)^25,30^. Samples of dorsal skin tissue at the injection site were collected before entry into the next HF phase (p.d. 3 and 18)^26^. For investigation of the telogen-anagen transition, mice were intradermally injected once daily with 100 μl PBS and morronside (100 μM, dissolved in PBS) on p.d. 0, 1, and 2 (for 3 days and a total of 300 μl) and sacrificed on p.d. 3. For analysis of the anagen-catagen transition, mice were administered the same volume and concentration of morroniside and PBS from p.d. 12 to 17 and sacrificed on p.d. 18 (for 6 days and a total of 600 μl). Skin samples were excised for analysis.

### Histological studies

The dorsal skin was fixed in 4% paraformaldehyde at 4 °C, embedded in paraffin, and then cut into sections at a thickness of 5–8 μm that were stained with hematoxylin and eosin (H&E). HF growth was evaluated according to four parameters: length, diameter of hair bulbs, HF percentage and HCS, and skin thickness^26^. At least 60 HFs per sample were analyzed. Based on previous studies, we assigned scores of 100, 200, and 300 for anagen I–IIIa, anagen IIIb–IIIc, and anagen IV–VI HFs, respectively, during telogen-to-anagen transition. We assigned scores of 100, 200, and 300 for anagen VI, catagen II–III, and catagen IV–V HFs, respectively, during anagen-to-catagen transition^25,26^.

### Immunohistochemistry

The dorsal samples were incubated with anti-β-catenin antibody. The primary antibody used in this study was rabbit anti-β-catenin (1:250; Cell Signaling Technology, Danvers, MA, USA). The results are expressed as the mean ± standard deviation (SD), and P < 0.05 was considered statistically significant.

### Statistical analysis

SPSS software (ver. 17.0; SPSS Inc., Chicago, IL, USA) was used to analyze the statistical significance, and all quantitative data are presented as the mean ± SD. All data were from at least three independent experiments. Student’s t-test and one-way ANOVA were used for comparisons of two groups and multiple groups, respectively. Asterisks denote statistical significance (*P < 0.05; **P < 0.01).

## Electronic supplementary material

Supplementary Information
