# Supplementary material for: Morroniside regulates hair growth and cycle transition via activation of the Wnt/β-catenin signaling pathway
Source: Sci Rep. 2018 Sep 13;8:13785. doi: 10.1038/s41598-018-32138-2 (PMC6137235; doi:10.1038/s41598-018-32138-2)
Supplement: Supplementary file 1 — Supplementary Information [file 41598_2018_32138_MOESM1_ESM.pdf]

Morroniside regulates hair growth and cycle transition via activation of the Wnt/β-catenin signaling pathway

Lijuan Zhou [#1], Han Wang [#1], Jing Jing[1], Lijuan Yu[1], Xianjie Wu[1], Zhongfa Lu[1, *]

Supplementary Figures

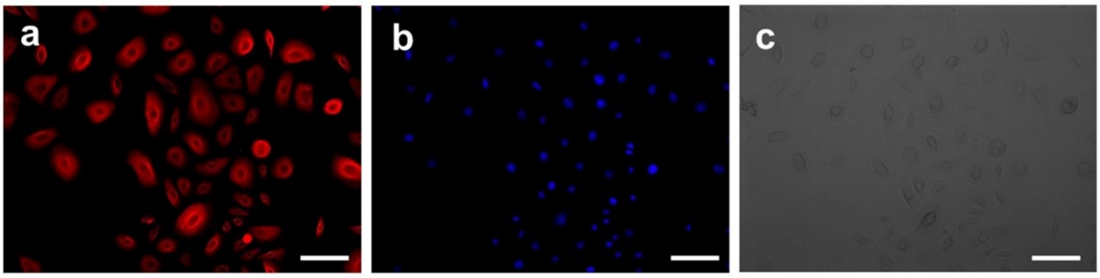

Supplementary Figure S1. Keratin 14 staining for ORSCs *in vitro*. a. ORSCs were stained for keratin 14 under a fluorescence microscope. b. ORSCs were stained for DAPI under a fluorescence microscope. c. ORSCs were observed under a normal microscope.

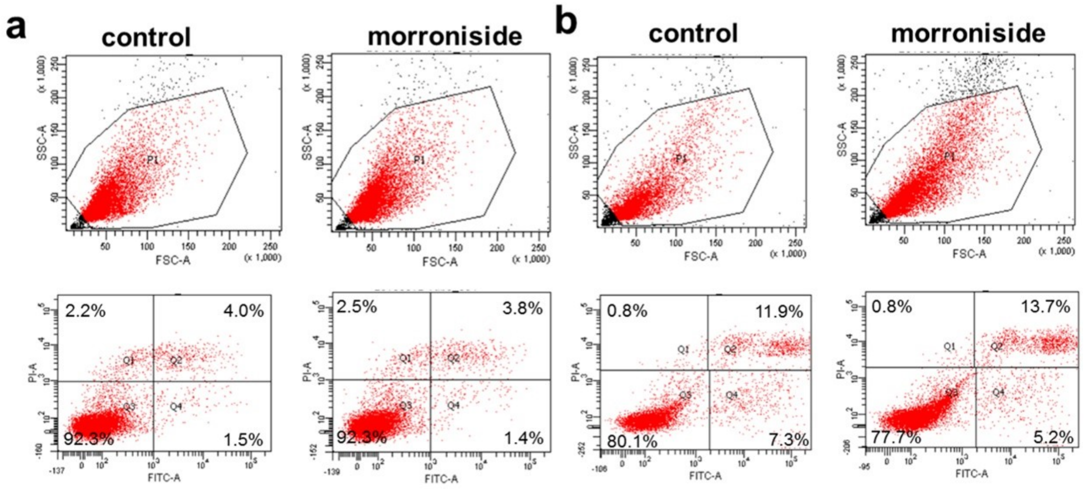

Supplementary Figure S2. Morroniside treatment had no significant effects on

ORSCs apoptosis in vitro. a-b. Representative pictures show the effect of morroniside treatment on ORSC apoptosis rate for 24 h (a) and 72 h (b) determined by flow cytometry. The morroniside-treated group showed no significant change compared with the control group after 24 h or 72 h.

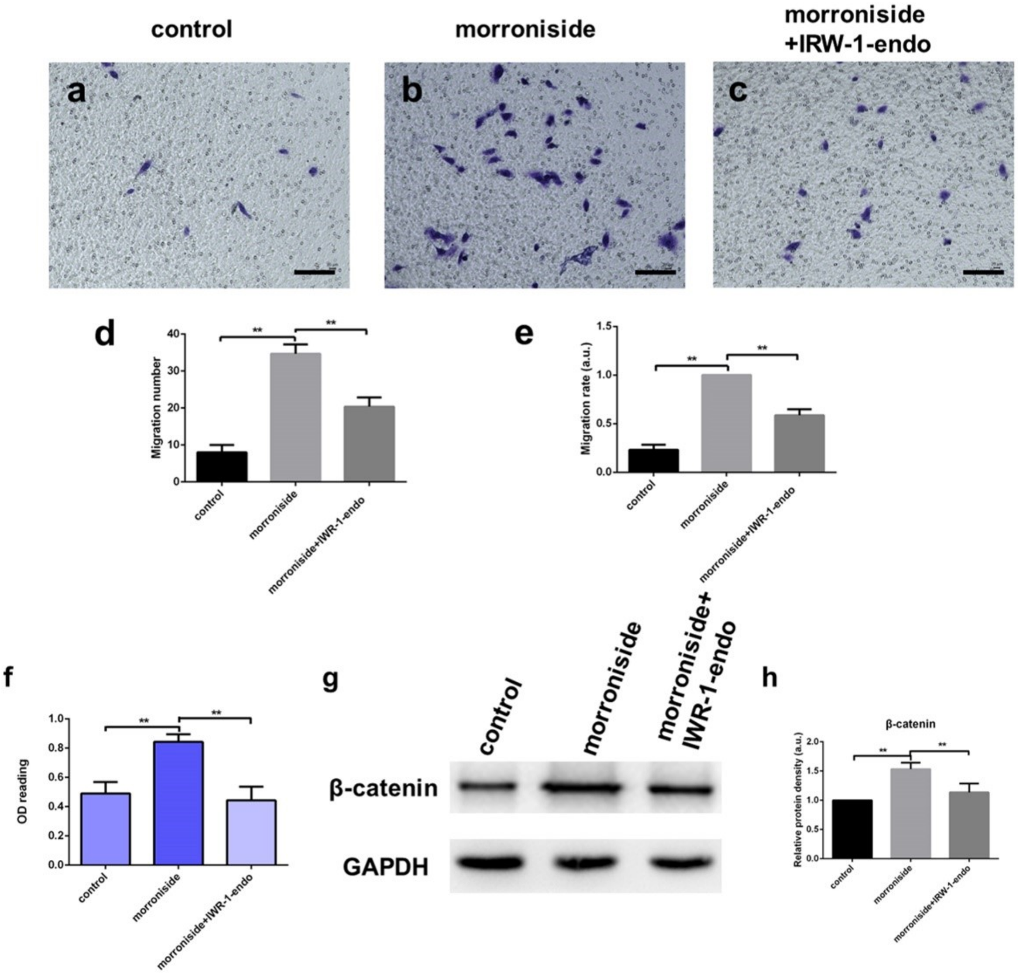

Supplementary Figure S3. The increased proliferation, migration, and β-catenin expression caused by morroniside were partially rescued by the addition of a Wnt/β-catenin signaling inhibitor (IRW-1-endo).

a-e. Transwell assays of ORSCs in control, morroniside and morronside + IRW-1-endo groups. **f.** The proliferation of ORSCs in the control, morroniside and morronside + IRW-1-endo groups was measured by MTS assays. **g-h. β**-catenin

protein levels in control, morroniside and morronside + IRW-1-endo groups were determined by western blotting. a.u., arbitrary units.
